# Compound Kushen injection combined with platinum-based chemotherapy for stage III/IV non-small cell lung cancer: A meta-analysis of 37 RCTs following the PRISMA guidelines

**DOI:** 10.7150/jca.40267

**Published:** 2020-01-20

**Authors:** Hongwei Chen, Xiaojun Yao, Ting Li, Christopher Wai-Kei Lam, Ruonan Zhang, Huixia Zhang, Jue Wang, Wei Zhang, Elaine Lai-Han Leung, Qibiao Wu

**Affiliations:** 1 State Key Laboratory of Quality Research in Chinese Medicines, Macau University of Science and Technology, Avenida Wai Long, Taipa, Macau 999078, China.; 2 Faculty of Medicine, Macau University of Science and Technology, Avenida Wai Long, Taipa, Macau 999078, China.; 3 Faculty of Chinese Medicine, Macau University of Science and Technology, Avenida Wai Long, Taipa, Macau 999078, China.

**Keywords:** Compound Kushen injection, platinum-based chemotherapy, non-small-cell lung carcinoma (NSCLC), systematic review, meta-analysis.

## Abstract

**Objective:** Compound Kushen injection (CKI), one of the commonly used antitumor Chinese patent medicines, has been widely prescribed as adjunctive treatment to platinum-based chemotherapy (PBC) in patients with advanced non-small cell lung cancer (NSCLC). However, the efficacy and safety of this combination therapy for advanced NSCLC remain controversial. The objective of this study is to evaluate the effects of CKI combined with PBC on patients with stage III/IV non-small cell lung cancer.

**Methods**: A systematic review and meta-analysis were performed following the PRISMA (Preferred Reported Items for Systematic Review and Meta-analysis) guidelines. All randomized controlled trials (RCTs) comparing CKI in combination with PBC versus PBC alone were retrieved and assessed for inclusion. Analyses were performed using Review Manager 5.3 (Copenhagen: The Nordic Cochrane Centre, The Cochrane Collaboration, 2014), Comprehensive Meta-Analysis 3.0 (Biostat, Englewood, NJ, United States; 2016) and Trial Sequential Analysis software (TSA) (Copenhagen Trial Unit, Centre for Clinical Intervention Research, Copenhagen, Denmark; 2011). The disease control rate (DCR) was regarded as the primary outcome, and the objective response rate (ORR), quality of life (QOL), survival rate, and toxicities were the secondary outcomes.

**Results:** Thirty-seven trials, recruiting 3,272 patients with stage III/IV NSCLC, were included. The results showed that, CKI combined with PBC resulted in significant improvements in DCR (RR = 1.11, 95% CI 1.07 to 1.15, P < 0.00001), ORR (RR = 1.30, 95% CI 1.20 to 1.40, P < 0.00001), QOL (RR = 1.73, 95% CI 1.55 to 1.92, P < 0.00001), 1-year survival rate (RR = 1.51, 95% CI 1.18 to 1.94, P = 0.001), and a 58% decline in the incidence of severe toxicities (RR = 0.42, 95% CI 0.37 to 0.49, P < 0.00001).

**Conclusions:** From the available evidence, our data indicate that CKI plus platinum-based chemotherapy is more effective in improving clinical efficacy and alleviating the toxicity of chemotherapy than platinum-based chemotherapy alone in the treatment of stage III/IV NSCLC. However, considering the intrinsic limitations of the included trials, high-quality RCTs with survival outcomes are still needed to further confirm our findings.

## Introduction

Worldwide, lung cancer remains the most common cancer and the leading cause of cancer-related mortality. In 2018, there were an estimated 2.1 million new lung cancer cases (11.6% of the total cancer cases) and 1.8 million deaths (18.4% of the total cancer deaths) 1-3. The incidence and mortality rates of lung cancer have significantly increased in recent years, and lung cancer has become a major public health problem in the developing world, including China 1, 4, 5.

Non-small cell lung cancer (NSCLC) accounts for approximately 85% of all lung cancer cases and about 66% of newly diagnosed NSCLC patients are already at stage III/IV 2. Over the past decade, targeted therapy and immunotherapy have been dramatically changing the therapeutic scenario and providing better outcomes to advanced NSCLC patients identified based on their molecular profiles. Unfortunately, not all patients can benefit from these precision therapies, due to lack of an actionable biomarker (more than 40% of NSCLC patients) or to unavailability of genomic testing and/or precision therapies, especially for the many patients in the developing world. For those patients, platinum-based chemotherapy (PBC) is still the cornerstone of treatment and a commonly recommended choice 6-10. Despite all efforts, the prognosis of stage IIIB/IV NSCLC remains extremely poor with a median survival of 7.9 months 11, and compared with precision therapies, the treatment with PBC alone is usually associated with worse survival, increased risk of toxic effects, and poor quality of life (QOL) 7, 9, 12. Therefore, there is a pressing need to improve the efficacy and safety of PBC for those patients who are treated with PBC rather than precision therapies.

In China and some Asian countries, traditional Chinese medicines have been increasingly prescribed for advanced lung cancer patients in combination with PBC for synergistic interactions 13, 14. Compound Kushen injection (CKI) is one of the commonly used anticancer Chinese patent medicines approved by the State Food and Drug Administration of China (Drug Approval Number: Z14021231) for the treatment of various cancers 15.

CKI is a mixture of natural compounds extracted from two medicinal herbs, Kushen (*Radix Sophorae* Flavescentis) and Baituling (*Rhizoma Smilacis* Glabrae). Active ingredients of CKI are matrine, oxymatrine, sophoridine, and N-methylcytisine 16, 17. Many studies have shown that CKI and its active ingredients have notable anti-tumor activities 17, 18, 19, such as inhibiting cancer cell proliferation, invasion and metastasis 16, 20, 21, inducing tumor cell apoptosis 22, 23, reducing angiogenesis 21, inducing cell cycle arrest 20, 21, 23, inhibiting glycometabolism and amino acid metabolism 16, and reversing multidrug resistance 24, 25. Besides, CKI can effectively increase immunologic function 26 and alleviate chemoradiotherapy-induced toxicity 21, 24, 25.

Emerging randomized controlled trials (RCTs) investigated the effects of CKI in combination with PBC on advanced NSCLC patients; however, the results were controversial. Some RCTs indicated that this combined therapy could improve the disease control rate (DCR), objective response rate (ORR), quality of life (QOL), and reduce adverse events 18, 19, but some trials found no significant changes in the above outcomes 27-32. The effects of CKI combined with PBC for patients with stage III/IV NSCLC have never been systematically assessed. Therefore, it is necessary to assess the efficacy and safety of CKI combined with PBC for patients with stage III/IV NSCLC, aiming to provide optimal therapy for the specific subsets of patients.

## Materials and Methods

We conducted this systematic review and meta-analysis following the PRISMA (Preferred Reported Items for Systematic Review and Meta-analysis) guidelines 33. This study has been registered with the International Prospective Register of Systematic Reviews (PROSPERO): CRD42019134892. Ethical approval was not required because the research materials were published studies.

### Types of Studies

All RCTs comparing CKI plus PBC versus PBC alone were selected and assessed for inclusion in this study.

#### Inclusion criteria

The participants included in this study should meet the following criteria: Diagnosis of stage III-IV NSCLC using the histopathological/cytological diagnostic criteria and TNM staging system 34-36 at least one bi-dimensionally measurable lesion; Karnofsky performance status (KPS) score 37 of at least 60; the range of Performance Status score from 0 to 2; life expectancy at least 3 months.

#### Exclusion criteria

The trials were not RCTs; sample size of either group was less than 30 patients; diagnosis was not NSCLC; the staging was not at stage III/IV; the NSCLC diagnosis was not specified accurately; the trials in which baseline data of the participants were inconsistent; there were no relevant outcome measures; the chemotherapy regimen was not platinum-based chemotherapy (PBC) or not clarified; radiotherapy, surgery, chemotherapy, immunotherapy, or Chinese medicine therapy other than CKI had been administered within three months before randomization; full-text articles or data was not available.

### Types of Interventions

Experimental group: CKI plus PBC; control group: PBC only. In each RCT, the PBC regimen used in both experimental and control groups was the same. All PBC regimens were eligible.

### Types of Outcome Measures

The primary clinical endpoint was the disease control rate (DCR). Objective response rate (ORR), quality of life (QOL), and toxic effects were defined as the secondary outcomes. According to the WHO criteria for reporting results of cancer treatment 38, 39, ORR and DCR were used to assess the short-term effectiveness. Improvement of QOL was considered when KPS score increased by 10 points or more after treatment. Anti-tumor drug toxicity was evaluated and classified as grades 0 to 4, according to Recommendations for Grading of Acute and Subacute Toxicity 38. In this research, grades 3 and 4 toxicities were defined as severe toxicities.

### Information Sources

A comprehensive literature search was conducted by two independent researchers (HW Chen and HX Zhang). Published studies were retrieved in common databases including PubMed, Web of Science, ClinicalTrials.gov, Cochrane Library, EMBASE, China National Knowledge Infrastructure (CNKI), Wanfang Databases, the Chinese Scientific Journal Database, the Chinese Science Citation Database, and the Chinese Biomedical Literature Database. The last search date was April 20, 2019. In addition, we searched and evaluated the relevant systematic reviews and meta-analyses to select the potential studies from their references.

### Search Strategy

The search details were conducted as follows (English database): {("Carcinoma, Non-Small-Cell Lung" [Mesh] OR "Carcinoma, Non-Small-Cell Lung" [Title/Abstract] OR "Carcinomas, Non-Small-Cell Lung" [Title/Abstract] OR "Lung Carcinoma, Non-Small-Cell" [Title/Abstract] OR "Lung Carcinomas, Non-Small-Cell" [Title/Abstract] OR "Non-Small-Cell Lung Carcinomas" [Title/Abstract] OR "Non-small Cell Lung Cancer" [Title/Abstract] OR "Non-Small-Cell Lung Carcinoma" [Title/Abstract] OR "Non-Small Cell Lung Carcinoma" [Title/Abstract] OR "Carcinoma, Non-Small Cell Lung" [Title/Abstract] OR "Non-Small Cell Lung Cancer" [Title/Abstract]) AND ("Compound Kushen injection" [Title/Abstract] OR "Fufang Kushen injection" [Title/Abstract] OR "Yanshu injection")}.Chinese databases (CNKI, etc.) searches: {"feixiaoxibaofeiai" ("carcinoma, non-small-cell lung") AND ("Fufangkushenzhusheye" ("Compound Kushen injection" OR "Fufang Kushen injection" OR "Yanshu injection") AND Hualiao ("chemotherapy")}.

### Study Selection

Two independent reviewers (HW Chen and T Li) screened all the candidate articles on the basis of title and abstract. The full texts were retrieved for further assessment according to the inclusion and exclusion criteria. All inclusion disagreements were resolved by consensus.

### Data extraction

Three reviewers (HW Chen, XJ Yao, and HX Zhang) independently rated the included RCTs and extracted the data. If a trial reported ambiguous or incomplete data, reviewers contacted the corresponding author via email and/or phone for further information. The intention-to-treat (ITT) analysis was used to analyze the results whenever available. The characteristics of all included RCTs are summarized in Table 1.

### Risk of Bias in Individual Trials

Two independent reviewers (HW Chen and T Li) appraised the risk of bias in the included trials using the Cochrane Risk of Bias Tool for Randomized Controlled Trials 38. The following criteria were used to evaluate bias in each trial: random sequence generation; concealment of allocation; blinding of participants and personnel; blinding of outcome assessment; incomplete data; selective reporting; and other bias. The risk of bias was classified as 'unclear', 'low' or 'high'.

### Summary Measures and Data Synthesis

All analyses were performed using the Review Manager (RM) 5.3 (Copenhagen: The Nordic Cochrane Centre, The Cochrane Collaboration, 2014), the Comprehensive Meta-Analysis (CMA) 3.0 (Biostat, Englewood, NJ, United States; 2016) and the Trial Sequential Analysis (TSA) software (Copenhagen Trial Unit, Centre for Clinical Intervention Research, Copenhagen, Denmark; 2011). Dichotomous data were shown as the risk ratio (RR), risk difference (RD) or odds ratio (OR), and continuous data were shown as the weighted mean difference (WMD) or standardized mean difference (SMD) with a 95% confidence intervals (CI). Heterogeneity was assessed using the *I*^2^ statistic and Chi^2^ test. Substantial heterogeneity was considered when *I*^2^ > 50% or *P* < 0.01. If the hypothesis of homogeneity was not rejected, a fixed-effects model was used to estimate the summary RR (OR or RD), WMD (or SMD) and 95% CI; otherwise, a random-effects model was used 40-43.

### Risk of Bias across trials

When the number of the included trials was ≥ 10, Egger's test and the funnel plots were used to examine the potential bias in the RCTs included in the meta-analysis 40, 44-46.

### Additional analyses

Sensitivity analysis, subgroup analysis and the Trial Sequential Analysis (TSA) were used to determine the robustness of results and calculate the required information size (RIS) in the meta-analysis 47. A meta-regression analysis was also carried out to examine the potential heterogeneity and whether the moderator variables have an impact on the study effect size.

## Results

### Study Selection

**Figure 1:**
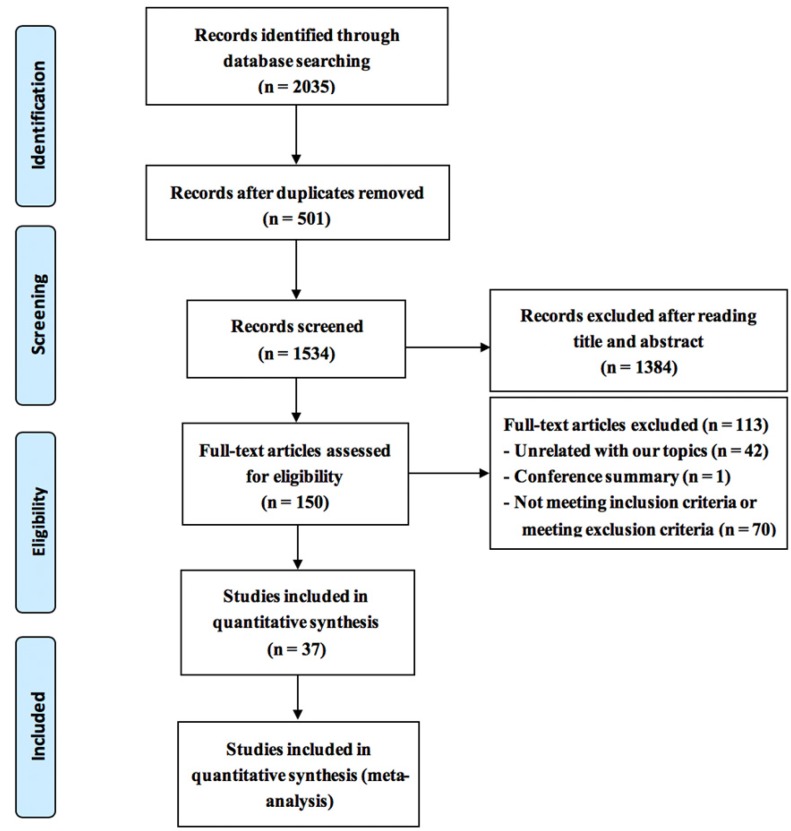
Preferred reporting items for systematic reviews and meta-analysis (PRISMA) search diagram. RCT = randomized controlled trial.

As shown in Figure 1, there were 2,035 records identified through the database search, 501 of them were duplicated and excluded. A total of 1,384 articles of case reports, reviews, letters, and basic researches were excluded after screening the title and the abstract. The full texts of 150 candidate papers were then screened and evaluated, and 113 were removed for the following reasons: unrelated with this research topic (n = 42); conference article (n = 1); not meeting inclusion criteria or meeting exclusion criteria (n = 70). Finally, 37 trials met the inclusion criteria. All the papers were in the Chinese language.

### Study Characteristics

Thirty-seven RCTs recruiting 3,272 patients were included (Table 1) 18, 19, 27-32, 48-76. All trials were conducted in China, and the articles were published from 2005 to 2017. All participants enrolled were patients with NSCLC at TMN stage III/IV. There were 1,670 and 1,602 patients in the experimental and control groups, respectively. The number of participants in each RCT varied from 60 to 144. The ages of the participants ranged from 18 to 80.

All RCTs included compared CKI combined with PBC versus PBC alone. Thirteen RCTs used TP (paclitaxel plus cisplatin), 10 RCTs used GP (gemcitabine plus cisplatin), 6 RCTs used NP (vinorelbine plus cisplatin), 2 RCTs used EP (Etoposide plus cisplatin), 1 RCT used DC (docetaxel plus carboplatin), 1 RCT used DP (docetaxel plus cisplatin), 1 RCT used TC (paclitaxel plus carboplatin), 1 RCT used PP (pemetrexed plus cisplatin) and 1 used RCT GC (Gemcitabine plus carboplatin), and 1 RCT used TP or GP regimens. In all experimental groups, CKI was synchronously administered with PBC for the patients. Twenty-five trials used 2 cycles of CKI for the patients in the experimental group, 8 trials used 3 cycles, and 4 trials used 4 cycles. The follow-up duration of the included RCTs varied from 1 to 30 months.

### Methodological quality

**Figure 2:**
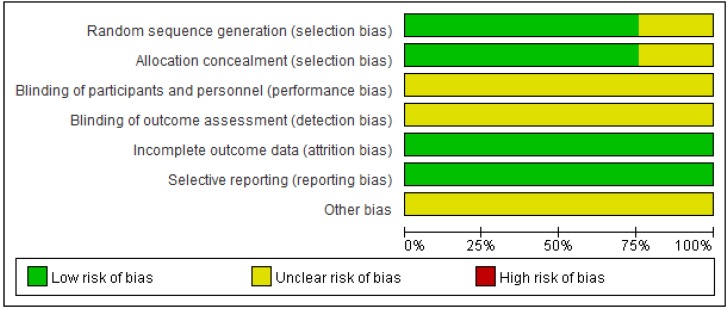
Risk of bias graph.

**Figure 3:**
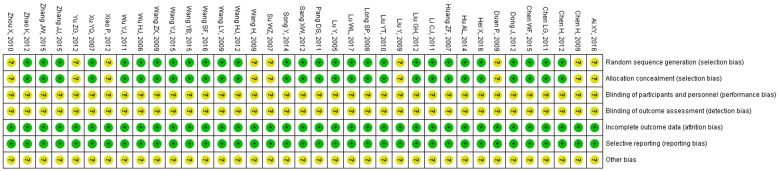
Risk of bias summary.

The risk of bias of all included RCTs was evaluated and summarized in Table 2, Figures 2 and 3. Randomization was used in all included studies, 28 studies clearly described appropriate randomization methods, risk of selection bias (random sequence generation) was low in these studies 18, 19, 27-29, 31, 50-52, 54-57, 59-62, 64-73, 75. Another 9 RCTs did not describe how the randomization was accomplished and the risk of selection bias was unclear 30, 32, 48, 49, 53, 58, 63, 74, 76. In all included RCTs, blinding of participants/personnel/outcome assessment was unclear. The data of all included trials were complete, the withdrawals and/or dropouts of patients were described and the reasons were reported. The risk of reporting bias was low. Any other bias was not clear.

### Outcome measures

The findings of the meta-analyses are summarized in Table 3.

#### Disease control rate (DCR) and objective response rate (ORR)

**Figure 4:**
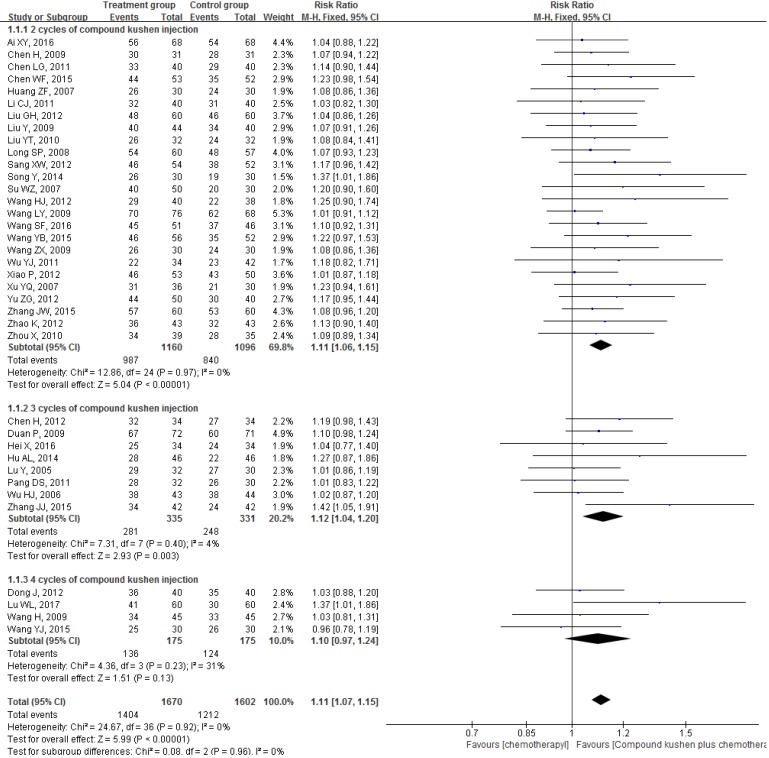
Forest plots showing a significant improvement in the DCR in the experimental group compared with that of the control group. CI: Confidence Interval; CKI: Compound Kushen injection, PBC: platinum-based chemotherapy.

**Figure 5:**
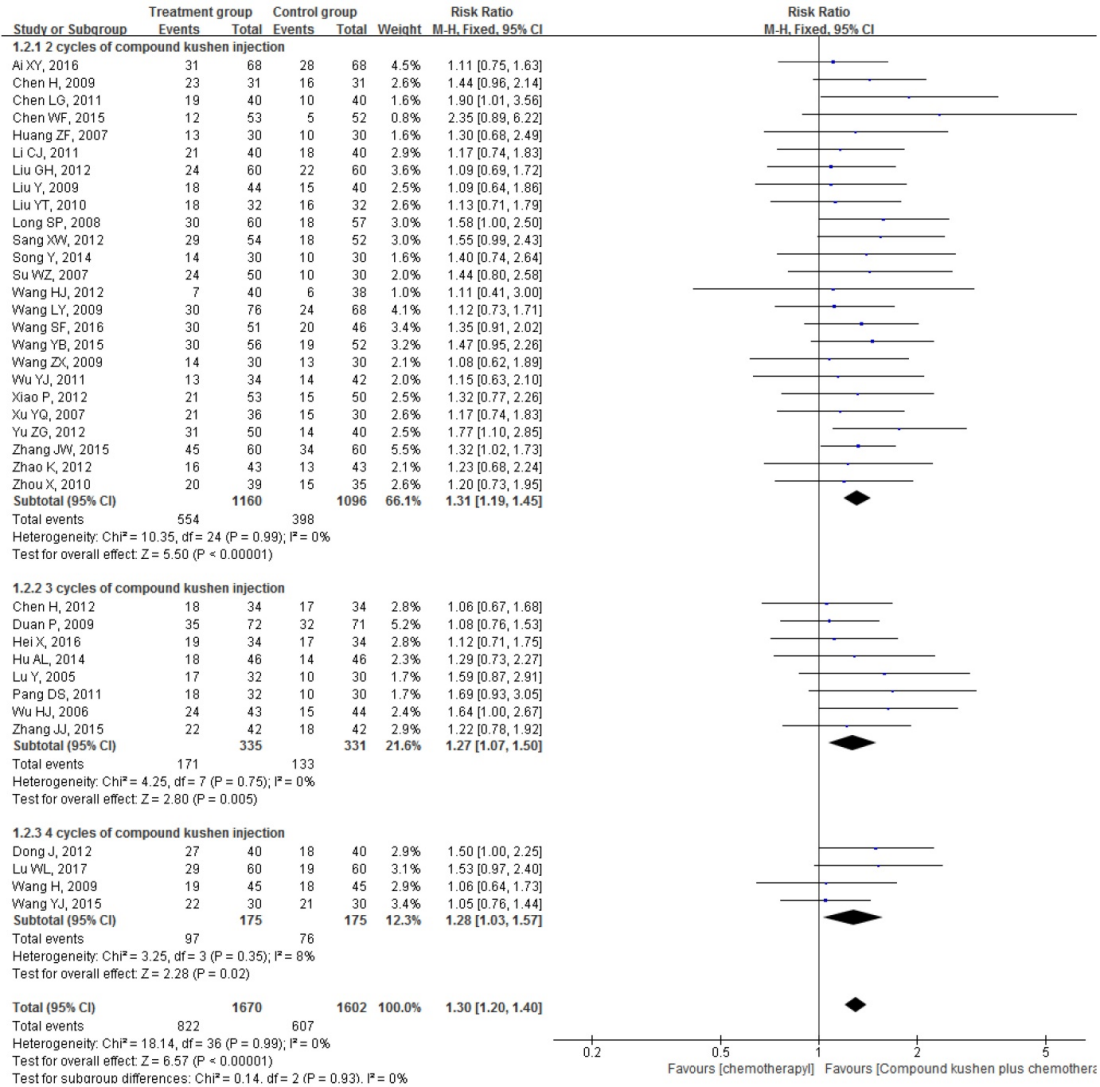
Forest plots showing a significant improvement in the ORR in the experimental group compared with that of the control group. CI: Confidence Interval; CKI: Compound Kushen injection, PBC: platinum-based chemotherapy.

All 37 included RCTs reported changes in DCR and ORR after the interventions. The pooled data showed that, compared with PBC alone, CKI plus PBC significantly improved DCR (RR = 1.11, 95% CI 1.07 to 1.15, *P* < 0.00001; Figure 4) and ORR (RR = 1.30, 95% CI 1.20 to 1.40, *P* < 0.00001; Figure 5). There was statistical homogeneity for both outcomes (both* I*² = 0%), and the fixed-effects model was used to combine the trials.

#### Improvement of QOL

**Figure 6:**
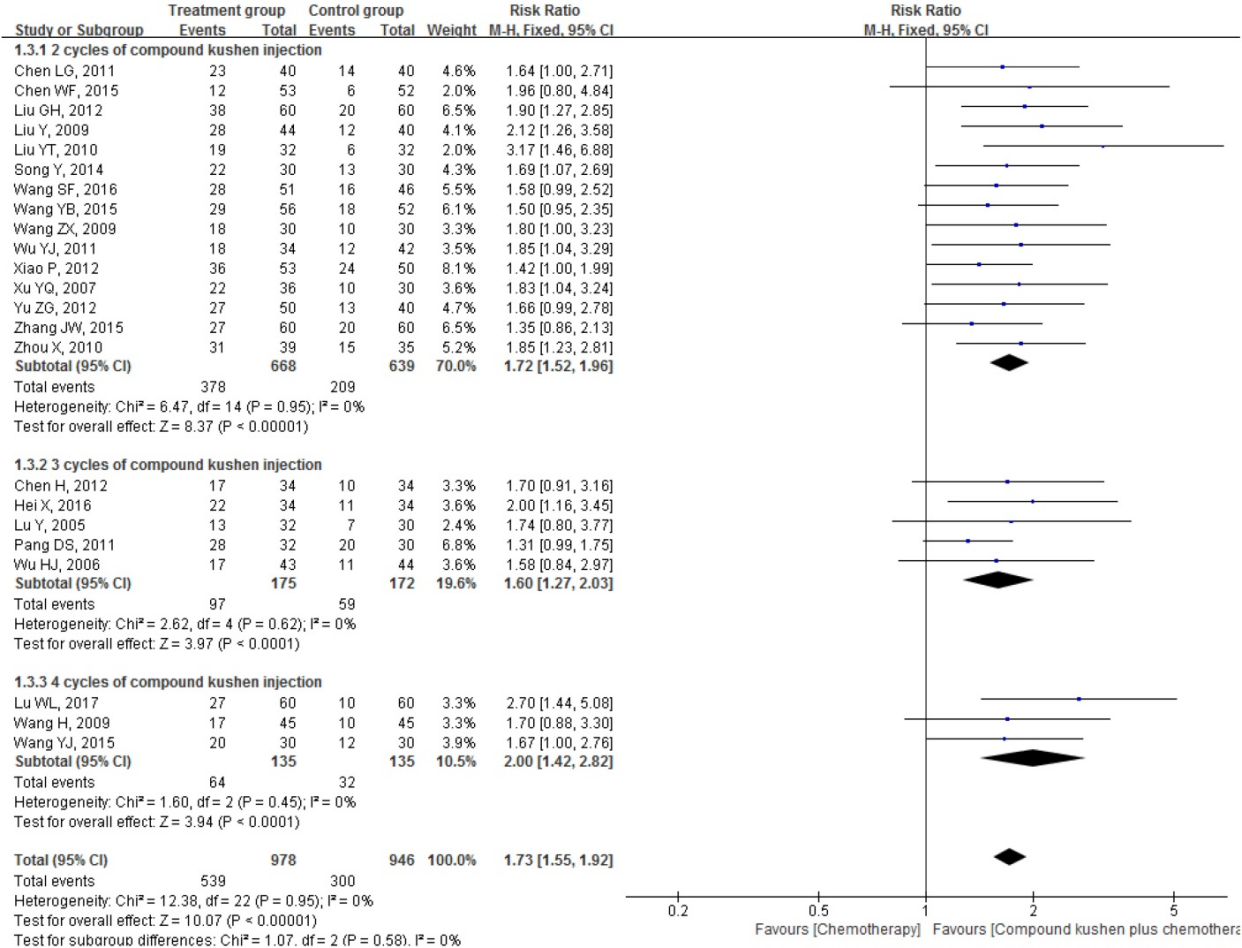
Forest plots showing a significant improvement in the QOL in the experimental group compared with that of the control group. CI: Confidence Interval; CKI: Compound Kushen injection, PBC: platinum-based chemotherapy.

Twenty-three trials investigated the effects of different interventions on the QOL 18, 19, 28, 29, 31, 32, 20-52, 53, 58-60, 64-67, 69, 70, 72-74, 76. Pooling data from these 23 studies showed that, compared with PBC alone, CKI plus PBC significantly improved QOL (RR = 1.73, 95% CI 1.55 to 1.92, *P* < 0.00001; Figure 6). There was statistical homogeneity for this outcome (*I*² = 0%), and the fixed-effects model was used to combine the trials.

#### Survival rates and other survival outcomes

**Figure 7:**
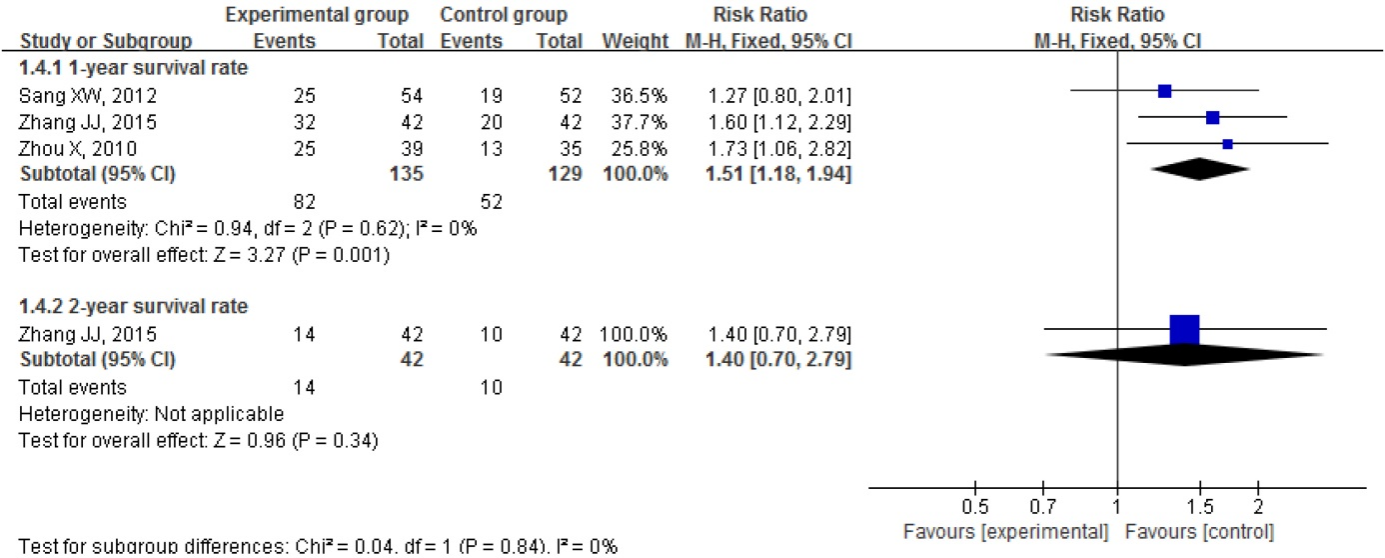
Forest plots showing a significant improvement in 1-year survival rate in the experimental group compared with that of the control group. CI: Confidence Interval; CKI: Compound Kushen injection, PBC: platinum-based chemotherapy.

Three trials reported the effect of CKI plus PBC on 1-year survival rate 62, 72, 76 and the result of meta-analysis showed that, compared with PBC alone, CKI plus PBC significantly increased 1-year survival rate (RR = 1.51, 95% CI 1.18 to 1.94, *P* = 0.001; Figure 7). There was statistical homogeneity for this outcome (*I*² = 0%), and the fixed-effects model was used to combine the trials.

Only one trial reported that there was no significant difference in the 2-year survival rate between groups (33.33% vs 23.81%, *P* > 0.05) 72.

Several RCTs reported four other survival outcomes including overall survival (OS) 32, progression-free survival (PFS) 31, 32, median time to progression (mTTP) 29, 62, 63, median survival time (MST) 29, 31, 62, 72, but, because of the unextractable data and/or the diversity of survival outcomes in the included RCTs, meta-analysis was not possible for these outcomes.

#### Toxicities

**Figure 8:**
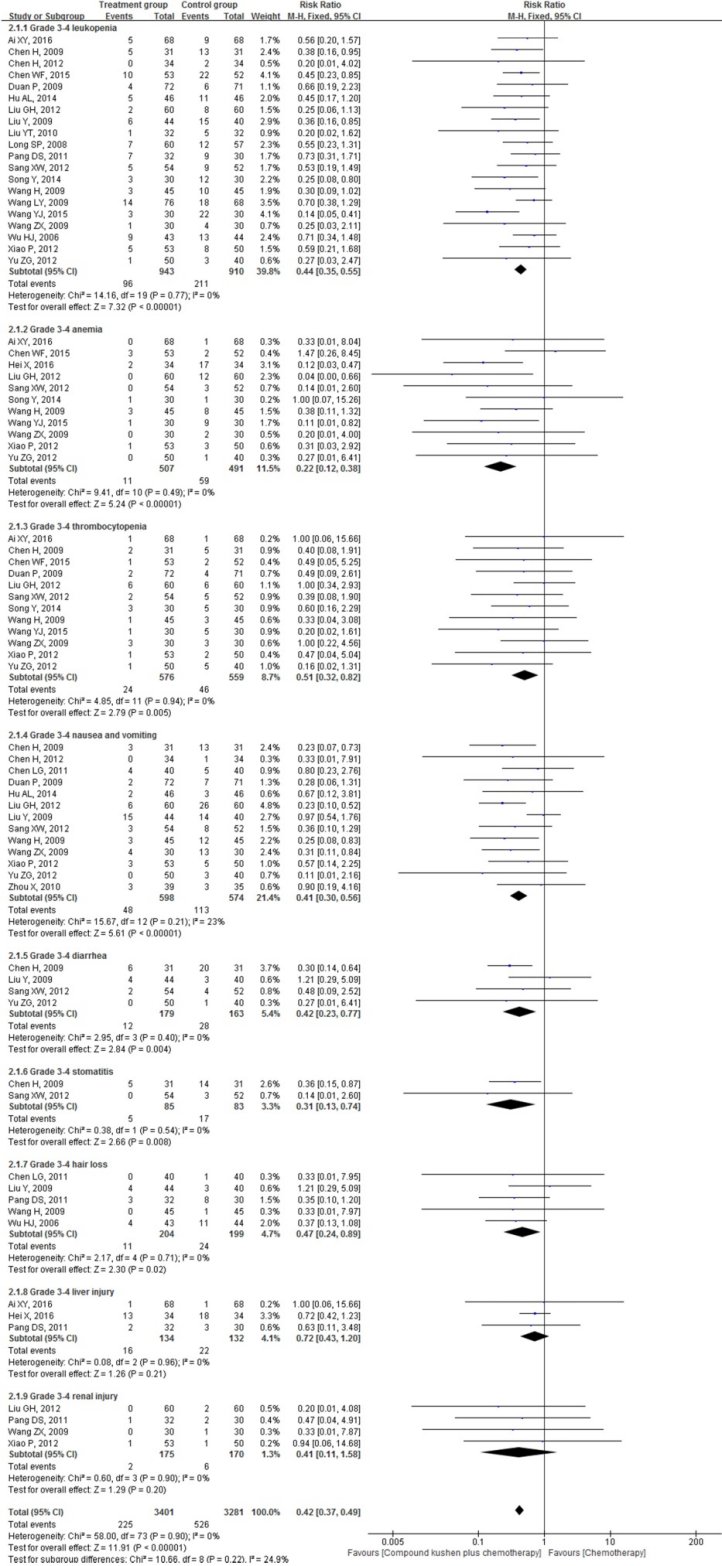
Forest plots showing a significant reduction of severe (grade 3 and 4) toxicities in the experimental group compared with those of the control group. CI: Confidence Interval; CKI: Compound Kushen injection, PBC: platinum-based chemotherapy.

Twenty-four RCTs reported the effect of CKI on severe (grade 3 and 4) toxic effects according to the WHO criteria 38. Compared with PBC alone, CKI plus PBC significantly reduced severe toxicities by 58% (RR = 0.42, 95% CI 0.37 to 0.49, *P* < 0.00001; Figure 8).

The findings of the meta-analysis demonstrated that CKI plus PBC was associated with significant reductions in severe leukopenia (RR = 0.44, 95% CI 0.35 to 0.55, *P* < 0.00001), anemia (RR = 0.22, 95% CI 0.12 to 0.38, *P* < 0.00001), thrombocytopenia (OR = 0.51, 95% CI 0.32 to 0.82, *P* = 0.005), nausea and vomiting (RR = 0.41, 95% CI 0.30 to 0.56, *P* < 0.00001), diarrhea (RR = 0.42, 95% CI 0.23 to 0.77, *P* = 0.004), stomatitis (RR = 0.31, 95% CI 0.13 to 0.74, *P* = 0.008), hair loss (RR = 0.47, 95% CI 0.24 to 0.89, *P* = 0.02). The differences between groups were statistically significant (Figure 8).

However, for severe liver injury (RR = 0.72, 95% CI 0.43 to 1.20, *P* = 0.21) and renal injury (RR = 0.41, 95% CI 0.11 to 1.58, *P* = 0.20), the differences between groups were not statistically significant (Figure 8).

There was no substantial heterogeneity for the above outcomes, and the fixed-effects model was used to combine the trials (Figure 8).

### Publication Bias

**Figure 9:**
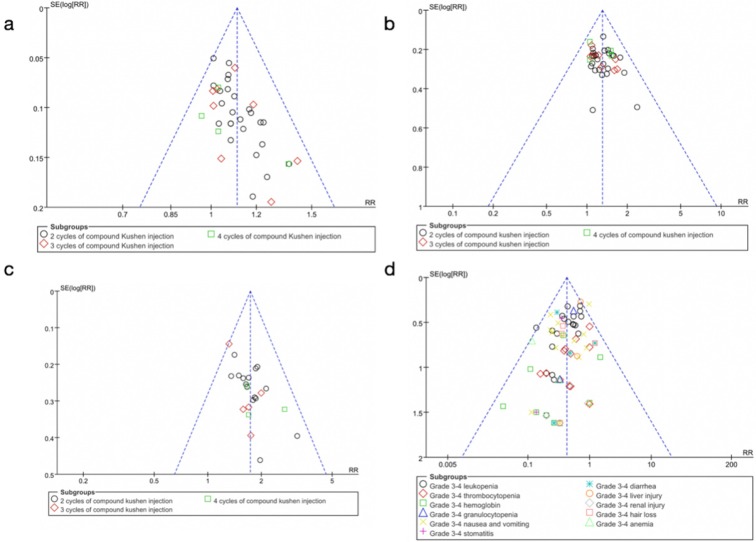
Funnel plots showing possible publication bias due to small-study effects. (A) Funnel plots of the DCR. (B) Funnel plots of the ORR. (C) Funnel plots of QOL. (D) Funnel plots of severe toxicities. RR: Relative Risk.

The funnel plots of the DCR suggested possible publication bias in small trials (Figure 9A), the Egger's test (*P* = 0.745) of DCR demonstrated that there was no obvious publication bias. The funnel plots of ORR (Figure 9B), QOL (Figure 9C), and severe toxicities (Figure 9D) also showed possible publication bias due to small-study effects.

### Subgroup and sensitivity analyses

With regard to DCR, the primary outcome, the pooled data showed that CKI plus PBC increased DCR significantly (RR = 1.11, 95% CI 1.07 to 1.15, *P* < 0.00001). Similar increases were observed when the subgroup and sensitivity analyses were performed based on the results of Cochrane Risk of Bias Tool (only including 28 RCTs with low risk of selection bias) (RR = 1.12, 95% CI 1.08 to 1.17, *P* < 0.00001) 18, 19, 27-29, 31, 50-52, 54-57, 59-62, 64-73, 75, participants number ( ≥ 50 in each group) (RR = 1.11, 95% CI 1.06 to 1.16, *P* < 0.0001), the cycle number of CKI [2 cycles (RR = 1.11, 95% CI 1.06 to 1.15, *P* < 0.00001), 3 cycles (RR = 1.12, 95% CI 1.04 to 1.20, *P* = 0.003), 4 cycles (RR = 1.10, 95% CI 0.97 to 1.24, *P* = 0.13)], PBC regimens [TP (RR = 1.13, 95% CI 1.06 to 1.21, *P* = 0.0002) , GP (RR = 1.11, 95% CI 1.05 to 1.18, *P* = 0.0004), NP (RR = 1.10, 95% CI 1.02 to 1.19, *P* = 0.003), EP (RR = 1.22, 95% CI 1.02 to 1.46, *P* = 0.03)], or publication year (only including the studies published within 5 years) (RR = 1.14, 95% CI 1.07 to 1.22, *P* < 0.0001) .

**Figure 10:**
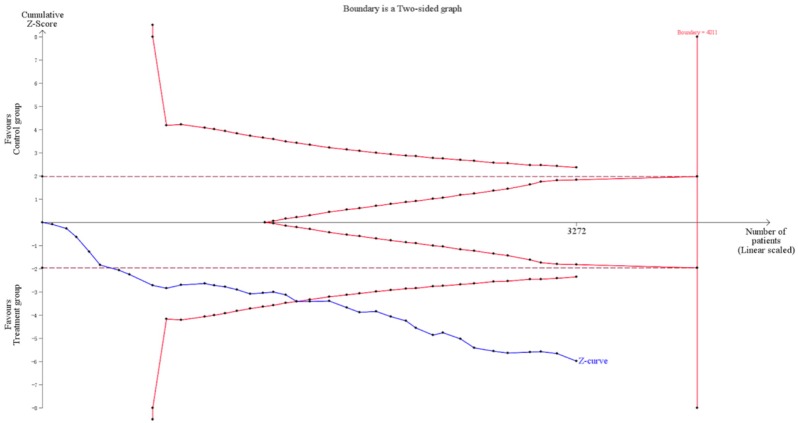
Trial sequential analysis of the DCR showing that the required information size had been reached and that Compound Kushen injection (CKI) plus PBC was significantly superior to the intervention in the control group. PBC, platinum-based chemotherapy.

**Figure 11:**
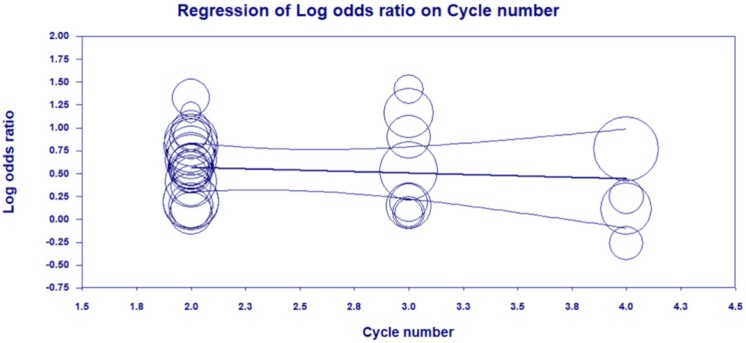
Meta-regression analysis showing that the DCR was not improved with an increased cycle number of Compound Kushen injection (CKI).

Trial sequential analysis (TSA) indicated the required information size for a reliable and conclusive meta-analysis had been reached, and that CKI combined with PBC was significantly superior to PBC alone (Figure 10), suggesting that the findings of the meta-analysis are robust for the DCR. The meta-regression analysis showed that the DCR was not improved with an increased cycle number (from 2 to 4) of CKI (Log OR = 0.543, *P* = 0.635; Figure 11).

For the ORR, QOL, and severe toxicity, the sensitivity and subgroup analyses showed similar results. Due to the limited number of included studies, the subgroup and sensitivity analyses were not available for the outcomes 1-year survival rate.

## Discussion

As an important anti-tumor drug in China, CKI is broadly used for treating many kinds of cancer. The efficacy and safety of CKI combined with chemotherapy for many cancers, such as esophageal cancer, breast cancer, hepatocellular carcinoma, colon cancer, etc. have been systematically evaluated 24, 25, 77. A previous meta-analysis evaluated the effects of CKI combined with radiotherapy for NSCLC, indicating that CKI plus radiotherapy significantly improved the clinical efficacy and reduced adverse events 78, however, the efficacy and safety of CKI plus PBC for advanced NSCLC patients have never been evaluated. The present study is the first systematic review and meta-analysis evaluating the effects of CKI plus PBC for advanced lung cancer patients. Our results showed that CKI as an adjunctive treatment to PBC can bring some crucial clinical benefits to the NSCLC patients at stage III/IV. This combined therapy could improve the tumor responses and QOL with a low risk of chemotherapy-induced toxicities in patients with advanced NSCLC. Our findings are consistent with the results of those meta-analyses evaluating the effects of CKI on other cancers 24, 25, 77, 79.

This meta-analysis included a total of 3,272 patients. Trial sequential analysis on DCR showed that CKI combined with PBC was significantly superior to PBC alone, and the findings of the meta-analysis are robust for the primary endpoint. The meta-regression analysis showed that the DCR was not improved with an increased cycle number (from 2 to 4), indicating 2 cycles of CKI might be an optimal treatment choice.

Both DCR and ORR are considered as important parameters for evaluating the short-term efficacy of antitumor therapy 14, 80-82. DCR, defined as the proportion of patients with complete response, partial response or no change, is regarded as the best response categorization to predict OS and progression-free survival (PFS). 80, 81 Therefore, it was defined as the primary clinical endpoint in our study. The pooled results of our meta-analyses clearly demonstrated that CKI plus PBC could significantly improve DCR (RR = 1.11, 95% CI 1.07 to 1.15) and ORR (RR = 1.30, 95% CI 1.20 to 1.40), suggesting that CKI increased the sensitivity of chemotherapy drugs and might also have synergic interactions with PBC. These are consistent with the results of previous meta-analyses evaluating the effects of CKI on the other cancers 24, 25, 77, 79, and the results of *in vitro* and *in vivo* experiments 24, 25. Because the drug sensitivity is a major concern in chemotherapy for NSCLC 83, 84, the benefits observed with the CKI for these two short-term outcomes are of important clinical significance.

The follow-up duration of the included RCTs was relatively short (1-30 months) though some trials investigated the long-term efficacy of CKI and reported various survival outcomes. Because of the limited number of the included trials reporting the survival outcomes and/or unextractable data, meta-analysis was only available for 1-year survival rate, and the result of meta-analysis shows that, compared with PBC alone, CKI plus PBC significantly increased 1-year survival rate. This study could not comprehensively assess the long-term efficacy of CKI for advanced NSCLC. In the future, it is necessary to include and investigate more survival outcomes in the clinical trials to further assess the long-term efficacy of CKI 84, 85.

The adverse reactions caused by chemotherapy, such as gastrointestinal reactions and myelosuppression, are very common, which may lead to chemotherapy discontinuation, poor QOL, and the situation where harm outweighs the benefits of PBC in many patients treated by chemotherapy. The subgroup meta-analysis indicated that CKI plus PBC could significantly reduce adverse reactions, such as nausea and vomiting, leukopenia, thrombocytopenia, hemoglobin, stomatitis, diarrhea, hair loss, and anemia, etc. Our results demonstrated that CKI combined with PBC was associated with a significant improvement in QOL (*P* < 0.00001), suggesting that combined treatment increases the tolerance to chemotherapy.

This meta-analysis has some limitations: all included studies were conducted in China and published in Chinese, some of the included studies were of the potential risk of bias, and the follow-up durations of all included trials were relatively short, etc. Therefore, more rigorous trials with longer follow-up periods are warranted to further assess the efficacy and safety of this combination therapy. Recently, a multicenter randomized controlled trial has been initiated to assess the efficacy of CKI in combination with chemotherapy in the treatment of elderly patients with advanced NSCLC 86. Such high-quality trials will help strengthen the evidence-base for this therapy 87.

## Conclusion

From the available evidence, our meta-analysis indicates that Compound Kushen injection combined with platinum-based chemotherapy is more effective in improving clinical efficacy and alleviating the toxicity of chemotherapy than chemotherapy alone for the treatment of stage III/IV NSCLC. However, high-quality RCTs with survival outcomes are still needed to further confirm our findings.

## Figures and Tables

**Table 1:** Principal characteristics of the studies included in the meta-analysis.

Reference	Design	Sample size (T/C)	Outcomes measure	Treatment group		Control group (Chemotherapy regimen)
				Intervention	Cycle number ofCKI plus PBC	
Ai XY, 2016 48	RCT	68/68	ORR, DCR, PFS, toxic effects	CKI + DP	2	DP
Chen H, 2012 51	RCT	34/34	ORR, DCR, QOL, toxic effects	CKI + TP	3	TP
Chen H, 2009 49	RCT	31/31	ORR, DCR	CKI + EP	2	EP
Chen LG, 2011 50	RCT	40/40	ORR, DCR, QOL, toxic effects	CKI + DC	2	DC
Chen WF, 2015 52	RCT	53/52	ORR, DCR, QOL, toxic effects	CKI + NP	2	NP
Dong J, 2012 27	RCT	40/40	ORR, DCR	CKI + PP	4	PP
Duan P, 2009 53	RCT	72/71	ORR, DCR, toxic effects	CKI + GP	3	GP
Hei X, 2016 54	RCT	34/34	ORR, DCR, QOL, toxic effects	CKI + TP	3	TP
Hu AL, 2014 55	RCT	46/46	ORR, DCR, toxic effects	CKI + TP	3	TP
Huang ZF, 2007 56	RCT	30/30	ORR, DCR	CKI + NP	2	NP
Li CJ, 2011 57	RCT	40/40	ORR, DCR	CKI + TP	2	TP
Liu GH, 2012 60	RCT	60/60	ORR, DCR, QOL, toxic effects	CKI + GP	2	GP
Liu YT, 2010 59	RCT	32/32	ORR, DCR, QOL, toxic effects	CKI + TP	2	TP
Liu Y, 2009 58	RCT	44/40	ORR, DCR, QOL	CKI + GP	2	GP
Long SP, 2008 61	RCT	60/57	ORR, DCR, toxic effects	CKI + TP	2	TP
Lu WL, 2017 18	RCT	60/60	ORR, DCR, QOL	CKI + GP	4	GP
Lu Y, 2005 28	RCT	32/30	ORR, DCR, QOL	CKI + EP	3	EP
Pang DS, 2011 29	RCT	32/30	ORR, DCR, QOL, toxic effects	CKI + TP	3	TP
Sang XW, 2012 62	RCT	54/52	ORR, DCR, QOL, 1-year survival rate, toxic effects	CKI + NP	2	NP
Song Y, 2014 19	RCT	30/30	ORR, DCR, QOL, toxic effects	CKI + GP	2	GP
Su WZ, 2007 63	RCT	50/30	ORR, DCR, toxic effects	CKI + NP	2	NP
Wang ZX, 2009 64	RCT	30/30	ORR, DCR, QOL, toxic effects	CKI + GP	2	GP
Wang H, 2009 30	RCT	76/68	ORR, DCR, toxic effects	CKI + TC	2	TC
Wang HJ, 2012 68	RCT	40/38	ORR, DCR	CKI + TP	2	TP
Wang LY, 2009 65	RCT	45/45	ORR, DCR, QOL, toxic effects	CKI + NP	4	NP
Wang SF, 2016 69	RCT	51/46	ORR, DCR, QOL	CKI + TP	2	TP
Wang YJ, 2015 66	RCT	30/30	ORR, DCR, QOL, toxic effects	CKI + TP	4	TP
Wang YB, 2015 67	RCT	56/52	ORR, DCR, QOL	CKI + TP	2	TP
Wu YJ, 2011 70	RCT	34/42	ORR, DCR, QOL	CKI + TP/GP	2	TP/GP
Wu HJ, 2006 31	RCT	43/44	ORR, DCR, QOL, toxic effects	CKI + NP	3	NP
Xiao P, 2012 32	RCT	53/50	ORR, DCR, QOL, toxic effects	CKI + GP	2	GP
Xu YQ, 2007 73	RCT	36/30	ORR, DCR, QOL	CKI + TP	2	TP
Yu ZG, 2012 74	RCT	50/40	ORR, DCR, QOL, toxic effects	CKI + GC	2	GC
Zhang JW, 2015 71	RCT	60/60	ORR, DCR, QOL	CKI + GP	2	GP
Zhang JJ, 2015 72	RCT	42/42	ORR, DCR, 1-year survival rate, 2-year survival rate	CKI + TP	3	TP
Zhao K, 2012 75	RCT	43/43	ORR, DCR	CKI + GP	2	GP
Zhou X, 2010 76	RCT	39/35	ORR, DCR, QOL, 1-year survival rate, toxic effects	CKI+ GP	2	GP

**CKI:** Compound Kushen injection, **DC**: Docetaxel plus carboplatin, **DCR**: Disease control rate, **DP**: Docetaxel plus cisplatin, **EP**: Etoposide plus cisplatin, **GC**: Gemcitabine plus carboplatin, **GP**: Gemcitabine plus cisplatin, **NP**: Vinorelbine plus cisplatin, **ORR**: Objective Response Rate, **PBC**: Platinum-based chemotherapy, **PFS:** Progression-free survival, **PP**: Pemetrexed plus cisplatin, **QOL**: Quality of life, **RCT**: Randomized controlled trial, **TC**: Paclitaxel plus carboplatin, **T/C**: Treatment group/control group, **TP**: Paclitaxel plus cisplatin.

**Table 2:** The methodologic quality of the included trials assessed using the Cochrane Risk of Bias Tool.

Reference	Random sequence generation	Allocation concealment	Blinding of participants and personnel	Blinding of outcome assessment	Incomplete outcome data	Selective reporting	Other bias
Ai XY, 2016 48	?	?	?	?	+	+	?
Chen H, 2009 49	?	?	?	?	+	+	?
Chen LG, 2011 50	+	+	?	?	+	+	?
Chen H, 2012 51	+	+	?	?	+	+	?
Chen WF, 2015 52	+	+	?	?	+	+	?
Dong J, 2012 27	+	+	?	?	+	+	?
Duan P, 2009 53	?	?	?	?	+	+	?
Hei X, 2016 54	+	+	?	?	+	+	?
Hu AL, 2014 55	+	+	?	?	+	+	?
Huang ZF, 2007 56	+	+	?	?	+	+	?
Li CJ, 2011 57	+	+	?	?	+	+	?
Liu Y, 2009 58	?	?	?	?	+	+	?
Liu YT, 2010 59	+	+	?	?	+	+	?
Liu GH, 2012 60	+	+	?	?	+	+	?
Long SP, 2008 61	+	+	?	?	+	+	?
Lu Y, 2005 28	+	+	?	?	+	+	?
Lu WL, 2017 18	+	+	?	?	+	+	?
Pang DS, 2011 29	+	+	?	?	+	+	?
Sang XW, 2012 62	+	+	?	?	+	+	?
Song Y, 2014 19	+	+	?	?	+	+	?
Su WZ, 2007 63	?	?	?	?	+	+	?
Wang LY, 2009 65	+	+	?	?	+	+	?
Wang H, 2009 30	?	?	?	?	+	+	?
Wang ZX, 2009 64	+	+	?	?	+	+	?
Wang HJ, 2012 68	+	+	?	?	+	+	?
Wang SF, 2016 69	+	+	?	?	+	+	?
Wang YJ, 2015 66	+	+	?	?	+	+	?
Wang YB, 2015 67	+	+	?	?	+	+	?
Wu HJ, 2006 31	+	+	?	?	+	+	?
Wu YJ, 2011 70	+	+	?	?	+	+	?
Xiao P, 2012 32	?	?	?	?	+	+	?
Xu YQ, 2007 73	+	+	?	?	+	+	?
Yu ZG, 2012 74	?	?	?	?	+	+	?
Zhang JJ, 2015 72	+	+	?	?	+	+	?
Zhang JW, 2015 71	+	+	?	?	+	+	?
Zhao K, 2012 75	+	+	?	?	+	+	?
Zhou X, 2010 76	?	?	?	?	+	+	?

+ = low risk of bias; ? = unclear risk of bias; - = high risk of bias.

**Table 3:** Summary of the meta-analysis (pooled data across categories in the control group).

Outcome or Subgroup	Studies	Participants	Statistical Method	Effect Estimate	*P*
DCR	37	3272	Risk Ratio (M-H, Fixed, 95% CI)	1.11 [1.07, 1.15]	< 0.00001*
			Odds Ratio (M-H, Fixed, 95% CI)	1.73 [1.45, 2.07]	< 0.00001*
			Risk Difference (M-H, Fixed, 95% CI)	0.08 [0.06, 0.11]	< 0.00001*
ORR	37	3272	Risk Ratio (M-H, Fixed, 95% CI)	1.30 [1.20, 1.40]	< 0.00001*
			Odds Ratio (M-H, Fixed, 95% CI)	1.62 [1.41, 1.87]	< 0.00001*
			Risk Difference (M-H, Fixed, 95% CI)	0.11 [0.08, 0.15]	< 0.00001*
QOL	23	1924	Risk Ratio (M-H, Fixed, 95% CI)	1.73 [1.55, 1.92]	< 0.00001*
			Odds Ratio (M-H, Fixed, 95% CI)	2.78 [2.29, 3.37]	< 0.00001*
			Risk Difference (M-H, Fixed, 95% CI)	0.23 [0.19, 0.27]	< 0.00001*
1-year survival rate	3	134	Risk Ratio (M-H, Fixed, 95% CI)	1.51 [1.18, 1.94]	0.001*
			Odds Ratio (M-H, Fixed, 95% CI)	2.35 [1.42, 3.88]	0.0008*
			Risk Difference (M-H, Fixed, 95% CI)	0.21 [0.09, 0.32]	0.0005*
Grade 3-4 toxicity	24	6842	Risk Ratio (M-H, Fixed, 95% CI)	0.42 [0.37, 0.49]	< 0.00001*
			Odds Ratio (M-H, Fixed, 95% CI)	0.35 [0.30, 0.41]	< 0.00001*
			Risk Difference (M-H, Fixed, 95% CI)	-0.09 [-0.11, -0.08]	< 0.00001*

**DCR**: Disease control rate, **ORR**: Objective Response Rate, **QOL**: Quality of life, **CI**: Confidence Interval.

## Abbreviations

CI: Confidence Interval
CKI: Compound Kushen injection
CMA: Comprehensive Meta-Analysis
CNKI: China National Knowledge Infrastructure
DC: Docetaxel plus carboplatin
DCR: Disease control rate
DP: Docetaxel plus cisplatin
EP: Etoposide plus cisplatin
GC: Gemcitabine plus carboplatin
GP: Gemcitabine plus cisplatin
ITT: Intention-to-treat
NP: Vinorelbine plus cisplatin
NSCLC: Non-small cell lung cancer
OR: Odds Ratio
ORR: Objective Response Rate
OS: Overall Survival
PBC: Platinum-based chemotherapy
PFS: Progression-free survival
PP: Pemetrexed plus cisplatin
QOL: Quality of life
RCT: Randomized controlled trial
RD: Risk Difference
RIS: Required information size
RM: Review Manager
RR: Risk Ratio
SMD: Standardized Mean Difference
T/C: Treatment group/control group
TC: Paclitaxel plus carboplatin
TP: Paclitaxel plus cisplatin
TSA: Trial Sequential Analysis
WHO: World Health Organization
WMD: Weighted Mean Difference
